# Immunosuppressive immune microenvironment landscapes in VISTA-high gastric cancer

**DOI:** 10.1038/s41416-025-03290-0

**Published:** 2026-01-26

**Authors:** Yiming Luo, Haoxin Peng, Qian Yao, Yi Xie, Dan Liu, Yakun Wang, Zhi Peng, Lin Shen, Yu Sun, Xiaotian Zhang, Yang Chen

**Affiliations:** 1 Department of Gastrointestinal Oncology, Key Laboratory of Carcinogenesis and Translational Research (Ministry of Education), Peking University Cancer Hospital and Institute, Beijing, China; 2 Department of Pathology, Key Laboratory of Carcinogenesis and Translational Research (Ministry of Education), Peking University Cancer Hospital and Institute, Beijing, China; 3 Early drug development center, Key Laboratory of Carcinogenesis and Translational Research (Ministry of Education, Beijing), Peking University Cancer Hospital and Institute, Beijing, China

**Keywords:** Tumour immunology, Gastric cancer

## Abstract

**Background:**

V-domain Ig-containing suppressor of T cell activation (VISTA) is an immune checkpoint molecule predominantly expressed on myeloid cells and has recently been recognised as a key mediator of immunosuppression within the tumour microenvironment (TME). However, its expression pattern in gastric cancer and the functional characteristics of the VISTA-high TME remain poorly understood.

**Methods:**

We conducted multiplex immunohistochemistry on tumour samples from 172 patients to characterise the immune landscape of the VISTA-high tumour microenvironment. Additionally, single-cell RNA sequencing (*n* = 17) and spatial transcriptomics (*n* = 3) were employed to delineate the cellular expression patterns of VISTA and investigate the potential immunomodulatory functions of VISTA-expressing macrophages.

**Results:**

High VISTA expression was associated with an immunosuppressive TME characterised by increased infiltration of exhausted CD8^+^ T cells, regulatory T cells (Tregs), M2-like macrophages, and cancer-associated fibroblasts (CAFs). Moreover, elevated VISTA levels in the tumour region were linked to worse immune-related progression-free survival (irPFS) in patients treated with immune checkpoint inhibitors (ICIs). Mechanistically, VISTA^+^ monocyte-macrophage (MoMac) populations promoted T cell exhaustion via the LGALS9-PTPRC signalling axis and exhibited enhanced antigen-presenting capacity.

**Conclusions:**

Our findings establish VISTA as a central immunoregulatory checkpoint in gastric cancer, suggesting its potential as a promising therapeutic target for combination immunotherapeutic approaches.

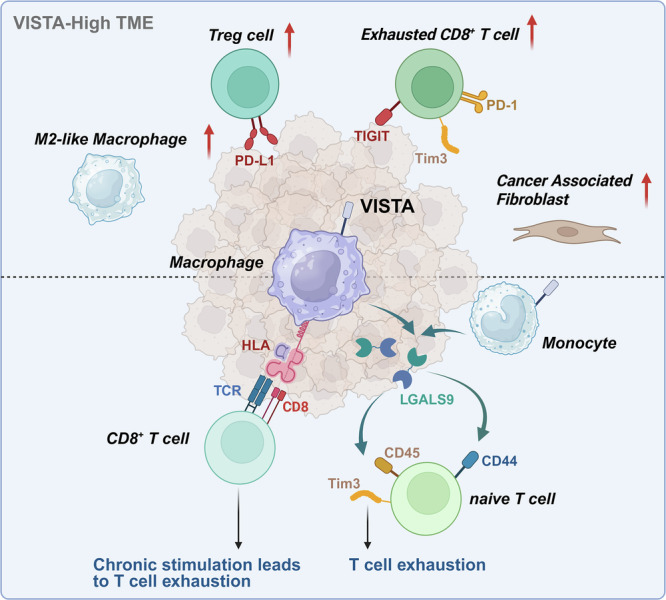

## Background

Gastric cancer (GC) remains a major global health challenge, ranking as the fourth leading cause of cancer-related mortality worldwide [1]. Advanced-stage GC is often accompanied by metastasis and limited treatment options, resulting in poor patient outcomes [1]. Despite advances in immune checkpoint inhibitors (e.g., pembrolizumab and nivolumab), the prognosis for advanced GC remains suboptimal, underscoring the need for novel therapeutic targets and precision medicine approaches [2].

VISTA (V-domain Ig-containing suppressor of T cell activation, B7-H5), a unique immune checkpoint molecule, has emerged as a key regulator of immune suppression within the tumour microenvironment (TME) [3]. VISTA is predominantly expressed on myeloid cells and functions as both a ligand and receptor, modulating T cell activation and myeloid cell polarisation [4, 5]. VISTA upregulation has been associated with immune escape in several malignancies, including GC, suggesting its potential as a therapeutic target [6]. Recent studies have highlighted the potential of VISTA in combination with PD-1/PD-L1 mAb to enhance the efficacy of immunotherapy. Preclinical models demonstrate that VISTA blockade restores T cell effector function and enhances antitumor immunity, particularly when combined with PD-1/PD-L1 inhibition [7]. Based on preclinical study results, a series of clinical trials targeting VISTA have been initiated, such as the anti-VISTA mAb SNS-101 in solid tumours (NCT05864144). Early-phase data suggest that VISTA blockade may overcome resistance to conventional ICIs, providing a rationale for its application in GC, where PD-1/PD-L1 inhibitor resistance remains a major clinical challenge [8].

Recent advances in multiplex immunohistochemistry (m-IHC), single-cell RNA sequencing (scRNA-seq), and spatial transcriptomics (ST) have provided powerful tools to dissect the TME in VISTA-positive GCs. Tumour-infiltrating myeloid cells, particularly macrophages expressing VISTA, have been implicated in promoting an immunosuppressive milieu [9]. Single-cell analyses have further identified distinct functional subsets of these cells and their interactions with other immune and tumour cells, offering mechanistic insights into their roles in immune evasion and therapy resistance. However, the functional roles and regulatory mechanisms of VISTA-positive immune cells within the TME remain poorly understood.

This study investigated the clinicopathological significance of VISTA expression in GC and its impact on immunotherapy outcomes. As a secondary aim, we sought to delineate the spatial patterns and immunological roles of VISTA-positive immune cells in the GC microenvironment through m-IHC and scRNA-seq.

## Methods

### Clinical cohort design

This study comprised three distinct cohorts of patients treated at the Department of Gastrointestinal Oncology, Peking University Cancer Hospital & Institute. Cohort A, a m-IHC cohort, included 172 patients with histologically confirmed gastric adenocarcinoma treated between January 2018 and November 2023. The primary objective of Cohort A was to investigate the TME characteristics of VISTA-positive gastric cancer. Cohort B, a single-cell sequencing cohort, consisted of 17 patients and aimed to analyse gene expression signatures of immune cell subtypes and tumour cells in VISTA-positive gastric cancer patients using scRNA-seq. Cohort C consisted of 3 patients and aimed to evaluate the spatial features of VISTA-related TME by ST. These cohorts were designed to provide complementary insights into the role of VISTA in gastric cancer progression and the tumour immune microenvironment.

Tumour samples and clinical data were collected and used with the guidance of the Declaration of Helsinki and approved by the Ethics Committee of Peking University Cancer Hospital (2023KT03). All participants signed informed consents.

### Tissue preparation and clinical outcome definitions

The tissue samples from each patient were preserved using the formalin-fixed, paraffin-embedded (FFPE) method. Patients with autoimmune diseases, HIV, or syphilis were excluded. This study was approved by the Peking University Cancer Hospital Ethics Committee, with informed consent obtained from all participants or their guardians. EBV status was determined using in situ hybridisation for Epstein–Barr encoded RNA 1 (EBER1). MMR status was assessed via IHC for DNA mismatch repair proteins (MLH1, MSH2, MSH6, PMS2) [10]. CPS was calculated as the percentage of PD-L1-positive tumour cells, lymphocytes, and macrophages [11]. According to the Response Evaluation Criteria in Solid Tumours (RECIST) version 1.1 (v1.1), responders were defined as those with complete or partial response, while non-responders had progressive or stable disease [12]. Overall-survival (OS) was defined as the time from diagnosis to death or end of follow-up, and immune-related OS (irOS) as the interval between immunotherapy start and death or last follow-up. Immune-related IrPFS was the time from immunotherapy start to progression, recurrence, death, or last follow-up.

### Multiplex immunohistochemistry

The m-IHC staining was performed to visualise the expression of CD8, PD-1, TIM-3, LAG-3, TIGIT, CD4, FoxP3, CTLA-4, PD-L1, CD68, CD163, CD86, STING, CD66b, SMA, FAP, CD54 and VISTA in tumour tissues in 4 panels. The specimens were collected within 30 min after tumour collection and fixed in formalin for 24–48 hours. Dehydration and paraffin embedding were performed using routine methods. Seven consecutive sections (4 µm-thick) were cut from paraffin blocks, one of which was used for H&E staining. Six FFPE tumour slides (4 µm) were melted at 60 °C for dehydration for 12 h. Paraffin sections were deparaffinized in xylene and rehydrated in alcohol. Heat-induced antigen retrieval was performed in ethylenediaminetetraacetic acid (EDTA) buffer, pH 9.0 (or citrate buffer, pH 6.0, for FoxP3 staining) using a microwave oven. The sections were blocked with commercially available blocking buffer (Dako, Santa Clara, CA; cat. X0909) for 10 min. The primary antibodies used for each staining are listed in Supplementary Table 1. The concentration and staining order of the antibodies used in this study were optimised in advance. The slides were serially incubated with primary antibodies and horseradish peroxidase-conjugated secondary antibodies (Biolynx, Hangzhou, China, cat. BX10001) and subjected to tyramide signal amplification (TSA). After each round of TSA operation, the slides were heated for antigen retrieval and antibody stripping. After all sequential staining steps, the cell nuclei were stained with 4′,6-diamidino-2-phenylindole (DAPI, Sigma-Aldrich, St. Louis, MO; cat. D9542) [13]. One experienced pathologist evaluated all stained GC specimens to ensure they met the further analysis requirements.

### Multispectral imaging

Imaging was conducted using the Mantra Quantitative Pathology Imaging System (PerkinElmer, Waltham, MA). Multiple 20× magnification fields were acquired within each tumour core for detailed analysis. Using the pathological analysis software QuPath, we developed an algorithm for tissue segmentation through a series of steps, including annotation, training, and validation. Specifically, PANCK-stained regions were classified as tumour regions, while non-PANCK-stained regions were classified as stromal regions. The algorithm was then applied to quantify and record the area and number of each tissue category (tumour and stroma) for each case, enabling precise and reproducible analysis of tissue composition in the samples. For standardisation, fixed-size stamps (930 × 700 µm, 20× objective lens) were applied in Phenochart (PerkinElmer) on previously acquired whole-slide images. To maximise coverage, viable regions within each specimen were selected with minimal overlap. Quality control (QC) was performed by a pathologist, who excluded unsuitable regions from analysis and verified any outlier data, ensuring robust dataset integrity.

### Recognition of cell morphology and spatial distribution

This study used inForm image analysis software to extract multispectral image features. Target proteins were visualised with fluorophores using antibodies on single-stained slides. A spectral library was created to extract autofluorescence spectra from unstained sections, which helped identify cellular phenotypes based on fluorophore properties and nuclear morphology (DAPI staining). The software analysed phenotypic features and classified DAPI-stained cells, quantifying the distribution and percentage of each cell type in tissue regions. Cellular spatial relationships were analysed by pairing central tumour cells with surrounding immune cells within a 20 μm radius, which was selected as a reasonable distance based on our previous research [14, 15]. Two metrics, effective score (immune cell-to-tumour cell ratio) and effective percentage (proportion of tumour cells paired with immune cells), were used to evaluate these networks:

$${{\rm{Effective}}}\; {{\rm{score}}}=\frac{{{\rm{number}}}\; {{\rm{of}}}\; {{\rm{paired}}}\; {{\rm{immune}}}\; {{\rm{cells}}}}{{{\rm{number}}}\; {{\rm{of}}}\; {{\rm{central}}}\; {{\rm{tumor}}}\; {{\rm{cells}}}}$$

$${{\rm{Effective}}}\; {{\rm{percent}}}=\frac{{{\rm{number}}}\; {{\rm{of}}}\; {{\rm{central}}}\; {{\rm{tumor}}}\; {{\rm{cells}}}\; {{\rm{with}}}\; {{\rm{paired}}}\; {{\rm{immune}}}\; {{\rm{cells}}}}{{{\rm{number}}}\; {{\rm{of}}}\; {{\rm{central}}}\; {{\rm{tumor}}}\; {{\rm{cells}}}}$$

These spatial dynamics offer insights into cell functions in the TME, which is crucial for understanding ligand-receptor-mediated interactions that influence antitumor or immunosuppressive effects.

### scRNA-seq sample collection and preparation

Fresh tumour tissues were preserved in GEXSCOPE® Tissue Preservation Solution (Singleron Biotechnologies, Nanjing, China) and transported on ice to the laboratory. After washing with Hanks’ Balanced Salt Solution (HBSS), tissues were minced into 1–2 mm pieces, digested with GEXSCOPE^®^ Tissue Dissociation Solution (Singleron Biotechnologies) at 37 °C for 15 minutes, and filtered through 40-µm strainers. The samples were centrifuged, treated with GEXSCOPE^®^ Red Blood Cell Lysis Buffer (Singleron Biotechnologies), and resuspended in phosphate-buffered saline (PBS). Finally, cell viability was assessed using trypan blue staining (Sigma-Aldrich, St. Louis, MO, USA).

### Generation of single-cell transcriptomic data

The sequencing data underwent processing through a customised computational workflow to generate cellular gene expression matrices. Briefly, raw reads were initially filtered to retain only those containing poly-T sequences, after which cellular identifiers and unique molecular tags were extracted. Adapter sequences and polyadenylated ends were trimmed using fastp (v1). Genomic alignment of Read 2 was performed against the GRCh38 reference genome with Ensembl 92 annotation, implemented through fastp 2.5.3a in conjunction with featureCounts 1.6.2 [16]. Transcript quantification was achieved by collapsing reads with matching cellular identifiers, molecular tags, and gene annotations to produce unique molecular identifier (UMI) counts for each gene-cell combination. The resulting UMI count matrices, organised by cellular identifiers, served as the foundation for downstream analytical procedures.

### Quality control, cell clustering, and annotation

The analysis of single-cell RNA-Seq data was conducted using the Seurat package (version 5.0.1, available at http://satijalab.org/seurat/) with a series of well-defined steps and parameter settings [17, 18]. Initial quality control was performed to filter out low-quality cells based on the following criteria: nFeature_RNA > 200 and < 5000, nCount_RNA > 500, and the proportion of mitochondrial RNA was capped at 20%. To further ensure data quality, potential doublets were identified and removed using the scDblFinder package [19]. Technical noise was minimised while preserving biological variability through data normalisation using the *LogNormalize* method. The top 4000 most variable features were then selected using the variance stabilising transformation method. The resulting subset was scaled and centred, and principal component analysis (PCA) was performed using the default parameters. To ameliorate sample or patient-specific batch effects, the Harmony R package (v0.1.0) was applied to the Seurat object using the patient identifier as the grouping variable [20]. The resulting Harmony embedding was used to perform UMAP dimensional reduction, neighbour finding, and cluster finding with the first 50 dimensions and the resolution of 0.5. The main cluster annotation was achieved using a curated list of validated cell markers (Supplementary Table S7) [21]. After performing main cluster differentiation, we further subdivided each subgroup with a resolution of 0.5 and annotated the subgroups according to the cell atlas [22–25].

### Evaluation of the functional signatures and cell signatures

A commonly used gene set to describe T cell and macrophage/monocyte cell functions includes cytotoxicity, exhaustion, pro-inflammatory activity, Treg signature, M1 score, M2 score, pro-angiogenic activity, phagocytosis, antigen presentation, and complement generation (Supplementary Table S8) [24, 26, 27]. These gene set scores are calculated at the single-cell level using the UCell package [28]. Gene set scoring is employed to assess functional differences among various subtypes of T cells and macrophages/monocytes.

### Gene set enrichment and pathway analysis

For each cell subgroup, we calculated differentially expressed genes (DEGs) using the *FindAllMarkers* function (min.pct = 0.25, logfc.threshold = 1.0, *p*_val_adj < 0.05). Subsequently, Kyoto Encyclopaedia of Genes and Genomes (KEGG) and Gene Ontology (GO) enrichment analysis was performed using the MSigDB databases, including the categories of biological process, cellular component, and molecular function. This approach was employed to aid in characterising the functional profiles of the cell subgroups.

We then transformed the single-cell data into pseudo-bulk data, followed by Gene Set Variation Analysis (GSVA) of pathways in the KEGG and GO databases, and performed inter-group differential analysis using limma regression [29].

### Cell communication analysis

To analyse cell-cell communication, we used the CellChat package, which integrates ligand-receptor interactions and signalling networks [30]. Membrane-bound and secreted proteins were annotated across time-point-specific clusters. Interaction significance (*P*-value < 0.05) was calculated based on ligand-receptor pairs and the Seurat-normalised expression matrix, enabling dynamic signalling pathway analysis.

### Trajectory inference analysis

To explore the differentiation dynamics and transitions among distinct cell subtypes, pseudotime trajectory analysis was performed using Monocle2 [31]. Dimensionality reduction was achieved via the DDRTree method, leveraging significantly DEGs. Cell lineage trajectories were then inferred based on pseudotime using default parameters. Additionally, the BEAM test in Monocle2 was applied to identify dynamic gene expression changes along each branch of the trajectory. Additionally, we conducted supplementary validation using the Slingshot package for multi-branch pseudotime analysis, with branches visualised based on the UMAP plot [32].

### ST sequencing

GC tissues were preserved in paraffin, cut into 10-micron slices, and placed on Visium slides for ST analysis. After dehydration and H&E staining, sections were scanned at 20× magnification. Pathologists reviewed the slides to confirm diagnoses and label tissue regions. Libraries were prepared using 10× Genomics kits, and sequencing data were analysed with Spaceranger software. Low-quality spots (genes <200 per spot or detected in <3 spots) and mitochondrial gene levels were filtered out. The ST data exhibited high quality, with an average of 24,000–27,000 reads per spot, median gene counts ranging from 2445 to 3814, and median UMI counts between 3778 and 7,228 across all samples (Supplementary Table S14). Data were normalised, and clustered via PCA, and cell types were mapped using scRNA-seq integration. Specifically, we used the *AddModuleScore* function in Seurat to calculate enrichment scores for gene signatures of specific cell subsets (e.g., C3⁺ macrophages) or functional pathways (e.g., antigen presentation, immunosuppression) within each Visium spot. We assessed the spatial distribution of VSIR using Moran’s I index and Getis-Ord Gi^*^ hotspots analysis [33, 34]. Cell signature scores within each spot were quantified by the AddModuleScore function.

### Statistical analysis

The independent sample *t*-test, analysis of variance (ANOVA), Mann–Whitney *U* test, Kruskal–Wallis test, or Pearson correlation analyses were used to assess the association between tumour-infiltrating immune cells (TIICs) and clinicopathological characteristics. Kaplan–Meier method was used to estimate survival functions and the log-rank test to compare survival distributions. Statistical analysis and visualisation were performed using R version 4.4.2. All *P* values were two-tailed, and *P* <  0.05 was used to define statistical significance.

## Results

### Clinicopathologic Features of the m-IHC Cohort and the Association of VISTA Expression with Poor Immunotherapy Response

**Fig. 1:**
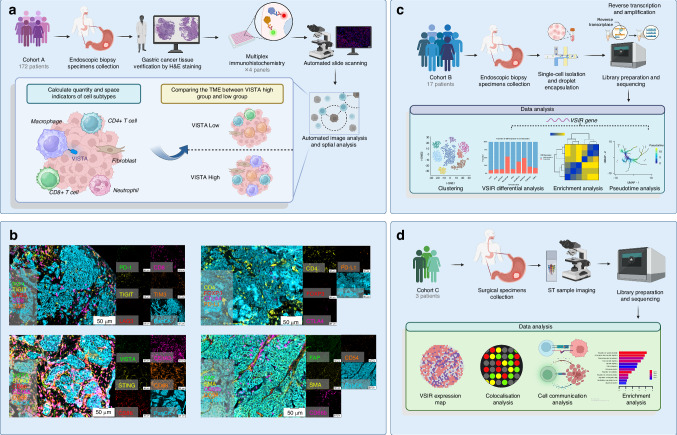
Identification and characterisation of the tumour microenvironment in GC patients according to VISTA expression. **A** Study flow chart and analysis design of the immunohistochemistry cohort. **B** The merged and single-stained images for 4 representative panels of multiplex IHC. Scale bar: 50 μm. **C** Study flow chart of single-cell RNA sequencing cohort. **D** Study flow chart of the spatial transcriptomics cohort. Created in BioRender.

**Fig. 2:**
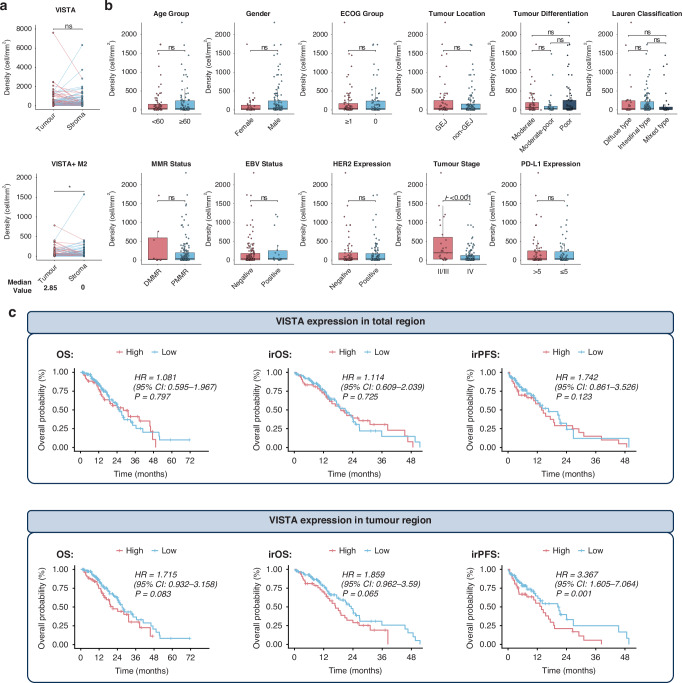
The association of VISTA expression with clinicopathological and prognostic features in GC. **a** The comparison of VISTA expression and VISTA^+^ M2-like macrophage density in Tumour/Stroma regions. Mann–Whitney *U* test. ns: not significant. **B** The association between VISTA expression in the total region and clinicopathological features. Mann–Whitney *U* test. ns: not significant. **C** Overall survival, immunotherapy related OS (irOS), immunotherapy related PFS (irPFS) of patients based on VISTA expression in total region and tumour region. The irOS and irPFS were calculated within immunotherapy-population. Log-rank (Mantel-Cox) test. HR: hazard ratio. HRs with 95% confidence interval (95% CI) and corresponding *P*-values were adjusted for the following covariates: age, ECOG performance status, tumour location, histological differentiation grade, Lauren classification, disease stage, HER2 expression status, PD-L1 expression level, MMR status, EBV status, and line of therapy.

In m-IHC Cohort, we included baseline pre-treatment samples from 172 patients, which were divided into four panels for staining (Fig. 1a, b). The median age of the patients was 62.00 years (range, 53.00–69.00), with 75.6% of the patients being male (Table 1). Eighteen (10.5%) patients had EBV-positive, and twelve (7.0%) had confirmed deficient DNA mismatch repair (dMMR) GC. We calculated the VISTA expression density in the total region (tumour + stroma) for each sample, defining the top 30% as high expression and the bottom 70% as low expression (cut-off value: 130 cell/mm²), and we conducted sensitivity analysis using different VISTA expression cut-offs to validate the robustness of our findings (Supplementary Fig. S3). Additionally, samples were categorised based on VISTA expression levels in the tumour and stromal regions (Supplementary Table S1, 2). Comparison between the tumour and stromal regions showed no difference in overall VISTA expression (Fig. 2a). However, the density of VISTA^+^ M2 macrophages (VISTA^+^ CD68^+^ CD163^+^ CD86^-^) was significantly higher in the tumour region than in the stromal region (*P* = 0.017) (Fig. 2a).

**Table 1:** The clinicopathological features between VISTA-high group and VISTA-low group based on Total regions.

Characteristic	All	High Group	Low Group	*P* value
	*N* = 172	*N* = 52	*N* = 120	
Age				0.326
median, IQR	62.00 [53.00, 69.00]	62.50 [53.75, 71.00]	62.00 [52.75, 68.00]	
Gender				0.396
Male	130 (75.6%)	42 (80.8%)	88 (73.3%)	
Female	42 (24.4%)	10 (19.2%)	32 (26.7%)	
ECOG PS				0.555
0	73 (42.4%)	26 (50.0%)	47 (39.2%)	
1	87 (50.6%)	24 (46.2%)	63 (52.5%)	
2	3 (1.7%)	1 (1.9%)	2 (1.7%)	
3	2 (1.2%)	0 (0.0%)	2 (1.7%)	
Unknown	7 (4.1%)	1 (1.9%)	6 (5.0%)	
Location				0.735
GEJ	58 (33.7%)	19 (36.5%)	39 (32.5%)	
non-GEJ	114 (66.3%)	33 (63.5%)	81 (67.5%)	
Differentiation				0.484
Moderate	57 (33.1%)	20 (38.5%)	37 (30.8%)	
Moderate-poor	39 (22.7%)	8 (15.4%)	31 (25.8%)	
Poor	73 (42.4%)	23 (44.2%)	50 (41.7%)	
Unknown	3 (1.7%)	1 (1.9%)	2 (1.7%)	
Lauren classification				0.676
Intestinal type	107 (62.2%)	33 (63.5%)	74 (61.7%)	
Diffuse type	27 (15.7%)	10 (19.2%)	17 (14.2%)	
Mixed type	35 (20.3%)	8 (15.4%)	27 (22.5%)	
Unknown	3 (1.7%)	1 (1.9%)	2 (1.7%)	
Stage				0.008
II	1 (0.6%)	1 (1.9%)	0 (0.0%)	
III	30 (17.4%)	15 (28.8%)	15 (12.5%)	
IV	138 (80.2%)	34 (65.4%)	104 (86.7%)	
Unknown	3 (1.7%)	2 (3.8%)	1 (0.8%)	
HER2 expression				0.307
Positive	102 (59.3%)	31 (59.6%)	71 (59.2%)	
Negative	69 (40.1%)	20 (38.5%)	49 (40.8%)	
Unknown	1 (0.6%)	1 (1.9%)	0 (0.0%)	
PD-L1 expression (CPS)				0.478
<1	42 (24.4%)	15 (28.8%)	27 (22.5%)	
1-5	28 (16.3%)	7 (13.5%)	21 (17.5%)	
5-10	24 (14.0%)	7 (13.5%)	17 (14.2%)	
>10	47 (27.3%)	17 (32.7%)	30 (25.0%)	
Unknown	31 (18.0%)	6 (11.5%)	25 (20.8%)	
MMR status				0.204
pMMR	153 (89.0%)	48 (92.3%)	105 (87.5%)	
dMMR	12 (7.0%)	4 (7.7%)	8 (6.7%)	
Unknown	7 (4.1%)	0 (0.0%)	7 (5.8%)	
EBV status				0.626
Positive	18 (10.5%)	7 (13.5%)	11 (9.2%)	
Negative	145 (84.3%)	43 (82.7%)	102 (85.0%)	
Unknown	9 (5.2%)	2 (3.8%)	7 (5.8%)	
Therapy Category				0.062
First-line	115 (66.9%)	31 (59.6%)	84 (70.0%)	
Second-line or Above	36 (20.9%)	10 (19.2%)	26 (21.7%)	
Neoadjuvant Therapy	21 (12.2%)	11 (21.2%)	10 (8.3%)	

*IQR* interquartile range, *GEJ* gastroesophageal junction, *dMMR* deficient mismatch repair, *pMMR* proficient mismatch repair.

The expression level of VISTA in stage III samples was significantly higher than in stage IV samples (Fig. 2b). However, no association was found between VISTA expression and age, gender, ECOG performance status, tumour location, tumour differentiation status, Lauren classification, HER2 status, PD-L1 status, or MMR status (Fig. 2b). Furthermore, we analysed the prognostic value of VISTA expression. There was no significant association between high VISTA expression in the total or stromal regions and prognosis (Fig. 2c, and Supplementary Fig. S1c). However, high VISTA expression in the tumour region was associated with poorer irPFS in the population receiving immunotherapy (HR = 3.367 (95% CI: 1.605–7.064), *P* = 0.001) (Fig. 2c).

### VISTA high expression correlates with a suppressive immune microenvironment

We analysed the differences in the immune microenvironment of GC samples with high VISTA expression, focusing on the comparison of expression differences in CD8^+^ T cells, CD4^+^ T cells, fibroblasts, macrophages, neutrophils, and various immune checkpoints.

**Fig. 3:**
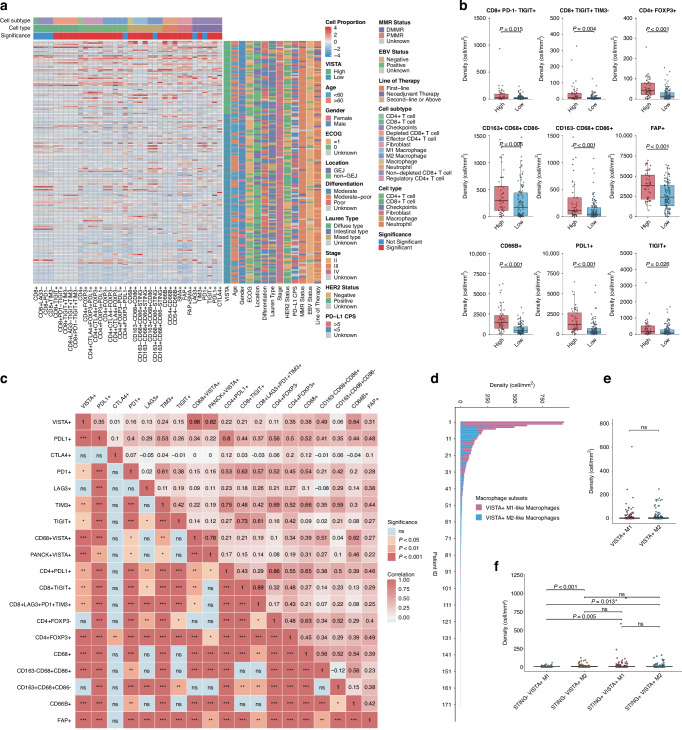
Expression patterns of unique TIICs in VISTA-high/low tissues. **a**, **b** The difference analysis of TIIC density between VISTA-high tissues and VISTA-low tissues. Significant: *P* < 0.05. Not Significant: *P* > 0.05. **c** Expression correlation between VISTA^+^ cells and other unique TIICs in the total region. ns: not significant. **d** Density of M1/2-like macrophages within total region in GC. **e**, **f** Comparison of the density of detailed macrophage subtypes among VISTA-positive macrophages in the total region. ns: not significant.

In the total region, no significant differences were observed in the density of non-exhausted CD8^+^ T cells between VISTA-high group and low group. However, a significant enrichment of CD8^+^ TIGIT^+^ associated T cell subsets (including CD8^+^ PD-1^-^ TIGIT^+^ T cells and CD8^+^ TIGIT^+^ TIM3^-^ T cells) was observed (Fig. 3a, b). Meanwhile, in the VISTA-high group, there was a significant increase in the expression of CD4^+^ cells, including both effector CD4^+^ T cells (including CD4^+^ FOXP3^-^, CD4^+^ FOXP3^-^ PD-L1^-^, and CD4^+^ FOXP3^-^ CTLA4^-^) and regulatory T cells (Treg cells, including CD4^+^ FOXP3^+^, CD4^+^ FOXP3^+^ PD-L1^+^, CD4^+^ CTLA4^+^ FOXP3^+^, and CD4^+^ CTLA4^+^ FOXP3^+^ PD-L1^+^) (Fig. 3a, b). In addition, M1-like macrophages (CD68^+^ CD163^-^ CD86^+^), M2-like macrophages (CD68^+^ CD163^+^ CD86^-^), fibroblasts (FAP^+^), neutrophils (CD66B^+^), and several immune checkpoints (including PD-L1 and TIGIT) were enriched in the VISTA-high group (Fig. 3a, b).

We further performed Pearson correlation analyses between VISTA expression and various immune checkpoints/cellular subpopulations in total, tumour and stroma regions (Fig. 3c, and Supplementary Fig. S4a, b). VISTA demonstrated strong positive correlations with VISTA⁺ macrophages (CD68⁺ VISTA⁺, r = 0.98) in total region and VISTA⁺ tumour cells in tumour region (VISTA⁺ PANCK⁺, r = 0.98) (Fig. 3c, and Supplementary Fig. S4a). Moreover, VISTA expression showed positive associations with FOXP3^+^ Treg cells (CD4^+^ FOXP3^+^  , r = 0.35, *P* < 0.001) and neutrophil (CD66B^+^, r = 0.64, *P* < 0.001) abundance, but not with FOXP3^-^ effector CD4^+^ T cells (CD4^+^ FOXP3^-^, *P* > 0.05) (Fig. 3c). Among immune checkpoints, PD-L1 exhibited the strongest correlation with VISTA expression (Total region: r = 0.35; Tumour region: r = 0.29; Stroma region: r = 0.47) (Fig. 3c, and Supplementary Fig. S4a, b). In summary, samples with high VISTA expression exhibited an inhibiting immune phenotype, where immune cells exerted immunosuppressive effects (such as exhausted CD8^+^ T cells, Treg cells, and M2 macrophages) in the VISTA-high GC TME.

Previous studies indicate predominant VISTA expression in myeloid cells within the TME, particularly macrophages [35]. We therefore quantified VISTA⁺ cell proportions across macrophage subtypes using m-IHC. While notable VISTA⁺ M1-like macrophage infiltration was observed in select specimens (Fig. 3d), comparative analysis revealed no statistically significant difference in cell densities between VISTA⁺ M1-like and VISTA⁺ M2-like macrophage populations (Fig. 3e). Given the emerging role of stimulator of interferon genes (STING) as a macrophage-associated pro-tumorigenic/immunosuppressive protein in GC, we evaluated STING-associated macrophage subsets [36]. Quantitative assessment demonstrated significantly reduced infiltration densities in VISTA⁺ M1 STING⁻ macrophages compared to other subsets (VISTA⁺ M1 STING⁺, VISTA⁺ M2 STING⁺, and VISTA⁺ M2 STING⁻). No significant inter-group differences were detected among the latter three subpopulations.

### Inhibiting spatial immune cell features in VISTA-High GC samples

**Fig. 4:**
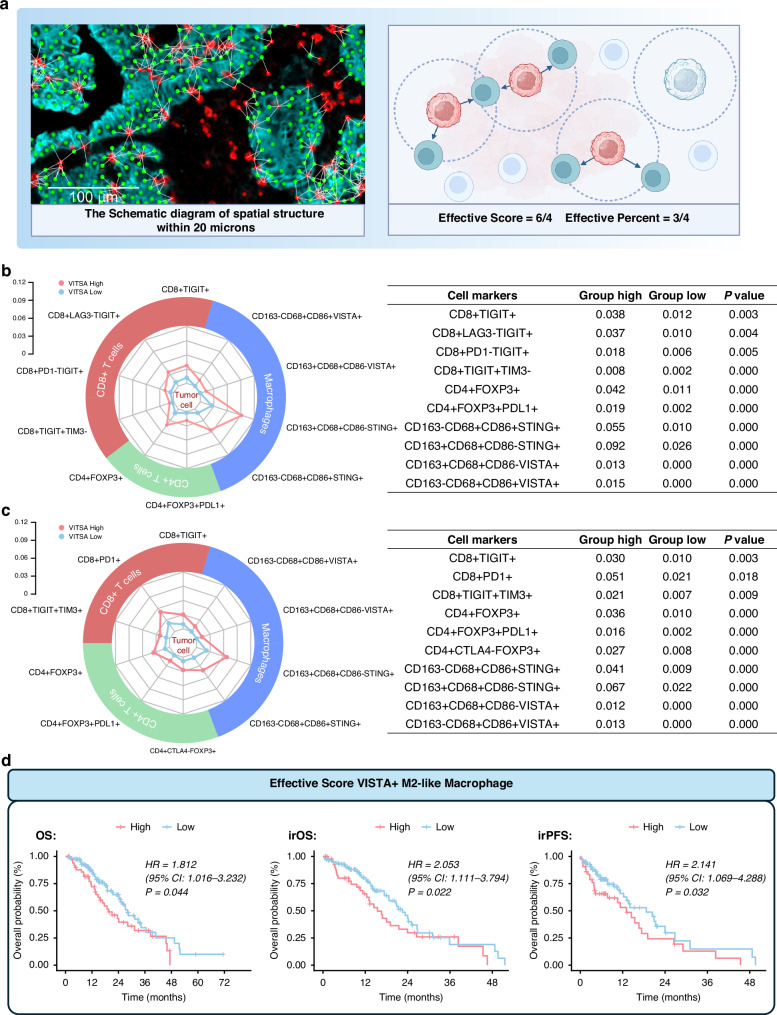
Spatial distribution of TIICs in VISTA-High GC. **a** Graphical depiction of spatial relationships between tumour and immune cells. Tumour cells are shown as green dots, immune cells as red dots, and connections via white lines indicate distances of less than 20 microns. **b** Differences in effective score were analysed between VISTA-high and VISTA-low groups. **c** Differences in effective percent were analysed between VISTA-high and VISTA-low groups. Mann–Whitney *U* test. **d** Kaplan-Meier survival analysis of VISTA^+^ M2-like macrophages stratified by effective score (high score defined as ≥70th percentile). Log-rank (Mantel-Cox) test. HR: hazard ratio. HRs with 95% confidence interval (95% CI) and corresponding *P*-values were adjusted for the following covariates: age, ECOG performance status, tumour location, histological differentiation grade, Lauren classification, disease stage, HER2 expression status, PD-L1 expression, MMR status, EBV status, and line of therapy. **a** Created in BioRender.

We used the developed spatial analysis tool to assess the spatial features in the VISTA-high group, focusing on differential analysis of the Effective score and Effective percent (Fig. 4a, b). Effective percent and Effective score calculate the densities and proportions of multiple immune cell types within a 20 μm radius of tumour cells (see Methods) [14].

The VISTA-high group exhibited significantly elevated effective score across multiple immune subsets compared to low group (Fig. 4b, and Supplementary Fig. S5a, b, Supplementary Table S4). Specifically, we observed marked increases in exhausted T cells (CD8⁺ TIGIT⁺: 0.038 vs. 0.012, *P* = 0.003), Treg cells (CD4⁺ FOXP3⁺: 0.042 vs. 0.011, *P* < 0.001), M1-like macrophages (CD163⁻ CD68⁺ CD86⁺ STING⁺: 0.055 vs. 0.010, P < 0.001), M2-like macrophages (CD163⁺ CD68⁺ CD86⁻ STING⁺: 0.092 vs. 0.026, *P* < 0.001), neutrophils (CD66b⁺: 0.758 vs. 0.299, *P* < 0.001), and fibroblasts (FAP⁺: 1.929 vs. 1.509, *P* = 0.001). Effective percent measurements demonstrated comparable patterns to absolute efficiency scores (Fig. 4c, and Supplementary Fig. S5b; Supplementary Table S5). In VISTA-high group, significant elevations of effective percent were observed across multiple immune subsets: exhausted T cells (CD8⁺ PD1⁺: 0.051 vs. 0.021, *P* = 0.018; CD8⁺ TIGIT⁺: 0.030 vs. 0.010, *P* = 0.003), Treg cells (CD4⁺ FOXP3⁺: 0.036 vs. 0.010, *P* < 0.001), M1-like macrophages (CD163⁻ CD68⁺ CD86⁺ STING⁺: 0.041 vs. 0.009, *P* < 0.001), M2-like macrophages (CD163⁺ CD68⁺ CD86⁻ STING⁺: 0.067 vs. 0.022, *P* < 0.001), neutrophils (CD66b⁺: 0.336 vs. 0.175, *P* < 0.001), and fibroblasts (FAP⁺: 0.638 vs. 0.567, *P* = 0.009).

We also investigated the relationship between the Effective score and the Effective percent of VISTA^+^ macrophages and the prognosis of GC patients. Patients were categorised into high and low groups based on the 70th percentile of the effective score and effective percent. Our results indicate that higher effective score/percent of VISTA^+^ macrophages correlate with worse irPFS in gastric cancer patients (score: HR = 3.036 (95% CI: 1.478 - 6.195), *P* = 0.002; percent: HR = 2.879 (95% CI: 1.409–5.881), *P* = 0.002), but no statistical association was found with OS/irOS (Supplementary Figs. S6a, b). We separately assessed the relationship between the Effective Score/percent of VISTA^+^ M1-like and VISTA^+^ M2-like macrophages and prognosis. The data showed that higher Effective score of VISTA^+^ M2-like macrophages was associated with worse OS (HR = 1.812 (95% CI: 1.016–3.232), *P* = 0.044), irOS (HR = 2.053 (95% CI: 1.111–3.794), *P* = 0.022), and irPFS (HR = 2.141 (95% CI: 1.069–4.288), *P* = 0.032) (Fig. 4d), while no significant association was observed for VISTA^+^ M1-like macrophages (Supplementary Figs. S6C, D).

### Single-cell RNA sequencing analysis of VSIR expression in specific monocyte-macrophage subsets in gastric cancer

**Fig. 5:**
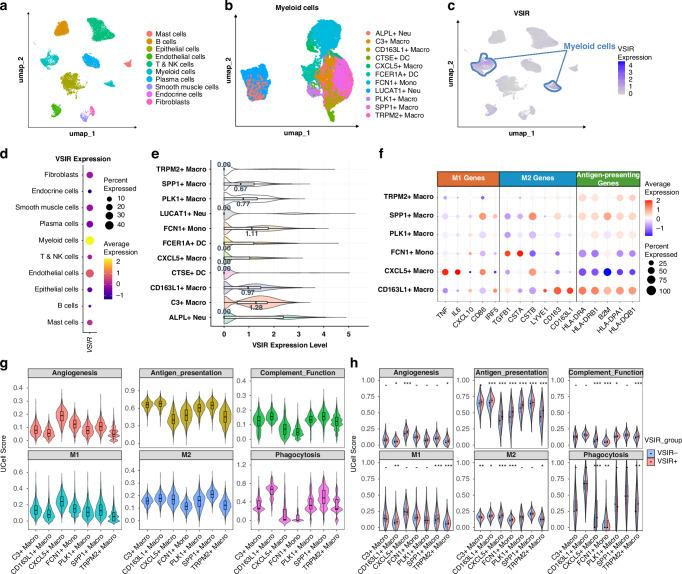
Single-cell transcriptomic atlas of GC patients reveals the expression landscape of VSIR across distinct cell populations. **a** Single-cell transcriptomic atlas of GC patients, showing distinct cell clusters including T&NK cells, B cells, plasma cells, myeloid cells, mast cells, endocrine cells, epithelial cells, endothelial cells, fibroblasts, and smooth muscle cells. **b** UMAP dimensionality reduction plots of myeloid cells subpopulations and the classification of various subsets. **c** VSIR expression mapped onto UMAP. Blue circles: high VSIR expression in myeloid cells. **d** The expression levels of VSIR across different cellular subpopulations were compared, revealing that its expression in myeloid cells was significantly higher than in other subpopulations. **e** Expression levels of VSIR in different myeloid cell populations, with the median values annotated in the bar chart. **F** Different subsets of Monocyte-Macrophage cells exhibit distinct characteristic M1/M2/antigen presentation functional genes. **g** Functional scores of multiple gene sets in monocyte-macrophage populations, calculated using UCell. **h** Comparison of gene set functional scores between VSIR^+^ and VSIR^-^ cells in monocyte-macrophage subpopulations. *P < 0.05, **P < 0.01, ***P < 0.001.

We performed scRNA-seq analysis on 17 GC samples collected from scRNA-seq Cohort. After quality control, we obtained 108,033 high-quality cells (Supplementary Fig. S7A). The cells were clustered into 27 subpopulations and annotated into 10 distinct subtypes: T & NK (Nature Killer) cells, plasma cells, B cells, myeloid cells, mast cells, fibroblasts, epithelial cells, endothelial cells, endocrine cells, and smooth muscle cells (Fig. 5A). The ROGUE scores of most cell subpopulations exceeded 0.7, while the ROGUE score for myeloid cells was relatively lower, suggesting the potential for further subdivision of the myeloid compartment (Supplementary Fig. S7C). We found that VSIR, the gene encoding VISTA, was prominently expressed in myeloid cells (Fig. 5C, D).

To further characterise the expression pattern of VSIR, we performed a finer subdivision of each cell category, with a particular focus on the myeloid compartment, which was further classified into macrophages, monocytes, dendritic cells (DCs), and neutrophils (Fig. 5b, and Supplementary Fig. S9a). The differential gene expression across subpopulations of myeloid cells is shown in Fig. 5e. We found that VSIR was primarily expressed in monocyte-macrophage (MoMac) subpopulations, including C3^+^ macrophages (median expression value: 1.28), FCN1^+^ monocytes (median expression value: 1.11), CD163L1^+^ macrophages (median expression value: 0.97), PLK1^+^ macrophages (median expression value: 0.77), SPP1^+^ macrophages (median expression value: 0.67), but not in DCs or neutrophils (Fig. 5e). We further scored the myeloid cells to assess the functional characteristics of various cell types within the MoMac subpopulation (Fig. 5g). The results revealed that VSIR was highly expressed in MoMacs with enhanced antigen presentation functions (C3^+^ macrophages and CD163L1^+^ macrophages) and M2-like macrophages (CD163L1^+^ macrophages and SPP1^+^ macrophages), rather than in the typical M1 macrophage subtypes (M1-like CXCL5^+^ macrophages) (Fig. 5f, g). We compared various functional scores between VSIR^+^ and VSIR^-^ cells within each MoMac subpopulation due to the expression pattern (Supplementary Fig. S10c). The results showed that VSIR^+^ cells in each subpopulation exhibited higher antigen presentation score(Fig. 5h, Supplementary Table S8). However, no significant differences were observed in the M1 score within the VSIR high-expression subpopulations (C3^+^ macrophages: 0.1311 vs. 0.1402, *P* = 0.528; FCN1^+^ monocytes: 0.1516 vs. 0.1484, *P* = 0.104). In conclusion, these findings indicate that VSIR is expressed in macrophages with enhanced antigen presentation functions in GC TME, rather than in the M1 macrophage subtypes.

### VSIR^+^ MoMac cells promote T cell exhaustion through the LGALS9-PTPRC pathway and antigen presentation

**Fig. 6:**
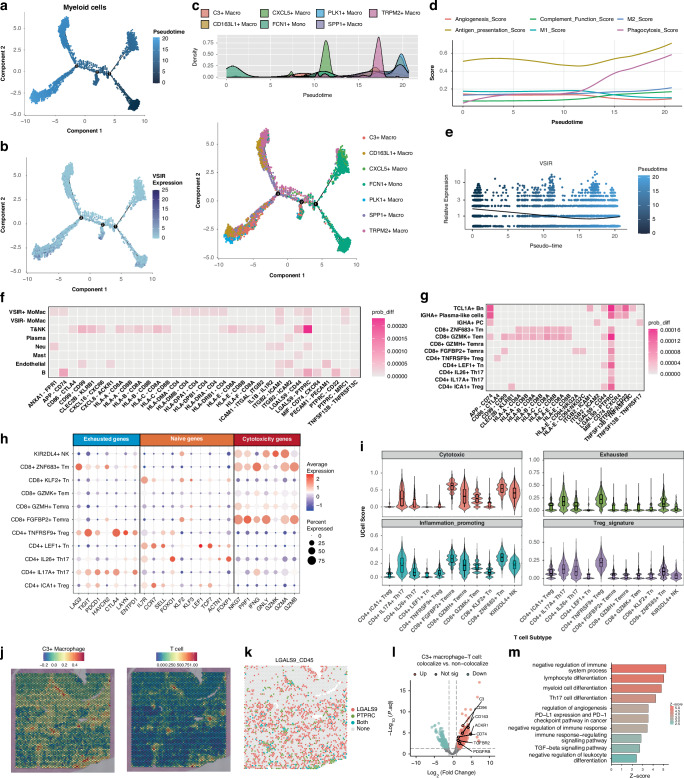
Pseudotime Analysis and Cell communications Analysis of VSIR^+^ monocyte-macrophages in GC TME. **a** Pseudotime trajectory depicting the dynamics of monocyte-macrophages (MoMacs). **b** Distribution of VSIR along the pseudotime trajectory in MoMacs. **c** Distribution of multiple MoMac cell subtypes along the pseudotime trajectory. **d** Dynamics of gene set functional scores along the pseudotime trajectory. **e** Identification of VSIR along the pseudotime trajectory. **f**, **g** Cell communication patterns upregulated and downregulated in VISR^+^ MoMacs compared to VSIR^-^ MoMacs with other cell subsets and various T cell subpopulations. Red indicates a higher probability of communication in VISR^+^ MoMacs, while blue indicates a lower probability. **h** Different subsets of T cells exhibit distinct characteristic naïve/exhausted/cytotoxcity functional genes. **i** Functional scores of multiple gene sets in T cell populations, calculated using UCell package. **J**. Spatial co-localisation of C3^+^ macrophages and T cells in ST1. **k** Inference of LGALS9-CD45 pathway distribution on spatial sections. **l**, **m** Differentially expressed genes (DEGs) and immune-related pathway enrichment analysis in co-localised regions of C3^+^ macrophages and T cells.

We performed differentiation trajectory analysis of MoMac cells using the Monocle and Slingshot packages, which yielded similar results (Fig. 6a, and Supplementary Fig. S11a). The differentiation trajectory revealed how MoMac cells differentiate from FCN1^+^ monocytes into various macrophage subpopulations, with a progressive shift toward the M2-like phenotype. During this process, antigen presentation and phagocytosis gradually increased (Fig. 6d). VSIR was prominently expressed in early-stage FCN1^+^ monocytes, early-to-mid-stage C3^+^ macrophages and late-stage CD163L1^+^ macrophages (Fig. 6b–e).

Additionally, we described the differences in cell communication between VSIR^+^ and VSIR^-^ MoMac cells. We found that VSIR^+^ MoMac cells predominantly interact with T & NK cells through the LGALS9 (Galectin-9)–PTPRC (CD45)/CD44 and HLA-CD8 pathways, and with B cells through the APP-CD74 pathway **(**Fig. 6f–h). We further examined the interactions between VSIR^+^ MoMac and T/B lymphocytes, scoring various T cell functions and performing temporal analysis to describe their roles (Fig. 6h, i, and Supplementary Fig. S12a–h). The results revealed that, compared to VSIR^-^ MoMac cells, VSIR^+^ MoMac cells preferentially interact with early-stage T cells (CD8^+^ GZMK^+^ Tem, CD4^+^ ICA1^+^ Treg, and CD4^+^ LEF1^+^ Tn) through the LGALS9–PTPRC/CD44 pathway **(**Fig. 6h, i). VSIR^+^ MoMacs also continuously stimulate CD8^+^ GZMK^+^ Tem and CD8^+^ ZNF683^+^ Tm cells through antigen presentation (Fig. 6G). The latter highly express PDCD1, LAG3, and HAVCR2, exhibiting characteristics of exhausted CD8^+^ T cells. This suggests that VSIR^+^ MoMacs may promote the differentiation of early CD8^+^ GZMK^+^ Tem into exhausted T cells through chronic antigen presentation [37]. Additionally, we stratified the samples based on the expression levels of VSIR in MoMac cells and found that in VSIR-high samples, the numbers of T cells, particularly CD4^+^ Treg cells and exhausted CD8^+^ T cells, were increased, which further demonstrated the role of VSIR in promoting T cell exhaustion (Supplementary Fig. S13c).

We further aimed to characterise VSIR-related expression and functional features using ST. In all three ST samples, VSIR exhibited a distinct nest-like spatial clustering pattern. C3^+^ macrophages spatially co-localised with T cells, suggesting potential interactions between the two cell types. Notably, the LGALS9-PTPRC interaction between C3^+^ macrophages and T cells was also validated in the ST data. Enrichment analysis revealed that C3^+^ macrophages were involved in both antigen presentation and immunosuppression. Interestingly, the dominance of these functions varied across samples: ST1 predominantly exhibited immunosuppression (Fig. 6q), ST3 showed primarily antigen presentation activity (Supplementary Fig. S14r), and ST2 displayed a balanced profile between the two (Supplementary Fig. S14k).

Overall, VSIR^+^ MoMac cells exhibit enhanced antigen presentation, which induced T cell exhaustion. And through LGALS9-related and APP-related pathways, they suppress the activation of early T cells and the functionality of cytotoxic T cells.

## Discussion

VISTA has traditionally been characterised as an immunosuppressive checkpoint; however, its varying prognostic implications across different cancer types have challenged this singular view. We assessed the immunological role of VISTA using multiple omics techniques, including m-IHC, scRNA-seq, and ST. In our comprehensive analysis of gastric cancer TME, findings suggest that VISTA functions as an inhibitory checkpoint. This study demonstrates that, beyond exerting classic immunosuppressive effects through its receptors and ligands, VISTA may engage in complex crosstalk within the TME, modulating T cell exhaustion.

Previous research has firmly established VISTA as a critical immune checkpoint with a unique expression pattern, predominantly on myeloid cells, and functioning as both a ligand and a receptor [38, 39]. In the context of gastric cancer, studies have confirmed its presence and association with an immunosuppressive Myeloid phenotype, often correlating with PD-L1 expression [6]. However, a comprehensive portrait of the VISTA-high TME has remained incomplete. We first characterised the immune microenvironment features associated with VISTA-high tumours, which represent immunosuppressive TME [40]. In the microenvironment with high VISTA expression, there is a greater enrichment of exhausted CD8^+^ T cells, Treg cells, CAFs, and M2-like macrophages. Additionally, the strong correlation between VISTA and other checkpoints (like PD-L1 and TIGIT) underlines the likely compensatory upregulation of alternative pathways in the setting of monotherapy, supporting the rationale for combinatorial immunotherapeutic strategies.

In our analysis of the relationship between VISTA and clinical characteristics, we observed that VISTA expression was higher in patients with Phase 3 compared to Phase 4, indicating that VISTA may not simply serve as a marker of poor immunotherapy response, which is consistent with previous studies [6]. In the prognostic analysis, we found that high expression of VISTA in the tumour region was associated with poorer irPFS. Distinct from previous studies that primarily classified samples based on VISTA expression levels without considering spatial context, we identified the prognostic significance of VISTA^+^ macrophage infiltration into the epithelial compartment. Using the spatial computational algorithm we previously developed [14, 15], we observed that the effective score of VISTA^+^ macrophages is associated with poorer irPFS. This suggests that VISTA is associated with poor immunotherapy outcomes, as the tumour microenvironment is enriched with more Treg cells and M2-like macrophages around tumour cells, which is likely the cause of the adverse effects. Furthermore, not only is there a significant increase in the number of these immune cells, but both the effective score and percent show that M2-like macrophages are significantly concentrated around tumour cells, outnumbering M1-like macrophages. Current clinical trials are also exploring the combination of PD-1 mAb and VISTA mAb to enhance the efficacy of immunotherapy; however, no results related to gastric cancer have been reported yet (NCT05082610, NCT05708950, NCT05864144). A gap remains between the prognostic relevance and the functional understanding of VISTA, as its high expression shows contrasting prognostic implications across different cancer types [4, 41, 42].

According to previous findings, VSIR^+^ macrophages appeared to exhibit a mixed phenotype, displaying both M1 and M2 characteristics [7]. However, the specific sub-typing characteristics of this population have not been clearly defined. Our study further demonstrates that VSIR is preferentially expressed in early-to-mid-stage differentiated MoMac cells and late-stage M2 macrophages, rather than in the late-stage M1 macrophage subtypes, and that VSIR+ macrophages possess enhanced antigen presentation functions. Compared to other macrophage immune checkpoints, the expression pattern of VISTA is quite unique. For instance, Signal Regulatory Protein Alpha (SIRPA) and Triggering Receptor Expressed On Myeloid Cells 2 (TREM2), which are typical inhibitory ligands predominantly expressed in terminal M2 macrophage subtypes, especially in SPP1+ macrophages [43, 44]. Additionally, the expression of VISTA varies considerably across different cancers. For example, in gastric cancer, multiple reports indicate its localisation to macrophages [6, 7], whereas in non-small cell lung cancer, VISTA is considered to be predominantly located in Treg cells [4].

Mechanistically, cell–cell communication results highlight that VSIR^+^ MoMac cells preferentially engage in antigen presentation with exhausted CD8^+^ T cells via the HLA-CD8 pathway. Recent studies suggest that persistent antigen presentation can drive the differentiation of pre-exhausted T cells into exhausted T cells, a process mediated by macrophages [37]. Combined with our findings, it can be proposed that VSIR^+^ MoMac cells promote T cell exhaustion through antigen presentation. We also observed an increase in exhausted T cells in VSIR-high samples, further supporting this notion. Notably, the LGALS9-CD45/CD44 pathway stood out, as VSIR^+^ MoMac cells were more inclined to communicate with early-phase T cells through this pathway. The LGALS9-CD44 pathway is believed to promote the function and proliferation of Treg cells [45], while the LGALS9-CD45 pathway is thought to collaborate with Tim-3, facilitating the exhaustion of CD8^+^ T cells [46]. This LGALS9-related interaction was validated in the ST analysis. Interestingly, we observed significant heterogeneity in the interactions between C3^+^ macrophages and T cells across different ST samples. However, this microenvironment does not seem to function through VISTA-mediated cellular communication. The ligands/receptors of VISTA—PSGL-1, VSIG-3, and LRIG1—could not be validated using the CellChat package, indicating the need for further understanding of VISTA’s role in macrophage regulation [39, 47].

While this study provides novel insights into the role of VISTA in GC, several limitations should be acknowledged. First, the m-IHC cohort (*n* = 172) provides sufficient power for robust immune profiling and survival analysis, consistent with similar gastric cancer immunology studies [14, 15]. However, the sample sizes for the scRNA-seq (*n* = 17) and ST (*n* = 3) cohorts were relatively small, which may limit the statistical power and generalisability of subgroup analyses. Notably, we observed significant heterogeneity in the functional polarisation of C3⁺ macrophages across the three ST samples. This sample-specific variation, while biologically intriguing, also reflects the limited sample size of our spatial cohort and warrants validation in larger studies. Second, the study was conducted at a single institution, potentially introducing selection bias. Multi-centre validation is required to confirm the prognostic and mechanistic associations observed. Third, while spatial and single-cell analyses revealed correlations between VISTA-expressing immune cells and clinical outcomes, functional experiments (e.g., in vitro/in vivo models) are needed to definitively establish causality and dissect the precise mechanisms by which VISTA modulates immune cell interactions. Finally, the study focused on baseline tumour samples; longitudinal analyses of dynamic changes in VISTA expression during immunotherapy could better elucidate its role in treatment resistance.

In conclusion, our study delineates the unique role of VISTA among immune checkpoints within the gastric cancer TME. Unlike conventional checkpoints such as PD-1/PD-L1, VISTA predominantly operates through specific myeloid cell subsets—particularly early-stage macrophages— to induce T cell exhaustion and establish an immunosuppressive niche via pathways such as LGALS9-PTPRC. The spatial and functional distributions of VISTA and its downstream effects diverge markedly from other checkpoints, highlighting the complexity and compensatory mechanisms of immune escape in GC. From a clinical perspective, our findings identify VISTA as a promising target for precision immunotherapy, especially for gastric cancer patients with high PD-L1 expression who nonetheless experience poor responses to current immune checkpoint inhibitors. The high VISTA expression signature marks a distinct subset of immunosuppressive TME, in which myeloid-driven suppression prevails despite active PD-L1 pathways. Targeting VISTA, alone or in combination with PD-1/PD-L1 blockade, could possibly overcome resistance mechanisms and expand the population of patients who benefit from immunotherapy. Our work provides a conceptual and translational framework for incorporating VISTA-targeted strategies into future clinical trials, ultimately aiming to optimise patient selection and improve outcomes in gastric cancer and potentially other solid tumours.

## Supplementary information

Supplementary Figures

Supplementary Tables

## Data Availability

The data generated and/or analysed during this study can be found in the supplemental information or from the authors upon request.
