# Supplementary material for: Immunosuppressive immune microenvironment landscapes in VISTA-high gastric cancer
Source: Br J Cancer. 2026 Jan 26;134(7):1066–79. doi: 10.1038/s41416-025-03290-0 (PMC12996324; doi:10.1038/s41416-025-03290-0)
Supplement: Supplementary file 1 — Supplementary Figures [file 41416_2025_3290_MOESM1_ESM.docx]

**Supplementary Figure S1.** The association of VISTA expression in tumor/stroma regions with clinicopathological and prognostic features in GC.

A. The association between VISTA expression in the tumor region and clinicopathological features. B The association between VISTA expression in the stroma region and clinicopathological features. C. Overall survival, immunotherapy related OS (irOS), immunotherapy related PFS (irPFS) of patients based on VISTA expression in stroma region. The irOS and irPFS were calculated within immunotherapy-population. Log-rank (Mantel-Cox) test. HR: hazard ratio. HRs and corresponding P-values were adjusted for the following covariates: age, ECOG performance status, tumor location, histological differentiation grade, Lauren classification, disease stage, HER2 expression status, PD-L1 expression level, MMR status, EBV status, and line of therapy.

**Supplementary Figure S2.** The difference analysis of TIIC density between VISTA-high tissues and VISTA-low tissues.

**A.** The difference analysis of overall TIIC density and checkpoints in total region between VISTA-high tissues and VISTA-low tissues. Significant: *P*<0.05. Not Significant: *P*>0.05. **B.** The difference analysis of overall TIIC density and checkpoints in tumor region between VISTA-high tissues and VISTA-low tissues. Significant: *P*<0.05. Not Significant: *P*>0.05. **C.** The difference analysis of overall TIIC density and checkpoints in stroma region between VISTA-high tissues and VISTA-low tissues. Significant: *P*<0.05. Not Significant: *P*>0.05.

**Supplementary Figure S3. Sensitivity analysis of VISTA expression cut-offs.**

**(A–C)** Comparisons between VISTA-high and VISTA-low groups, stratified using different expression cut-offs (Top 50%, 40%, and 25%). For each cut-off, analyses include: (i) differences in overall tumor-infiltrating immune cell (TIIC) density and immune checkpoint expression across the entire tissue region, and (ii) survival outcomes based on VISTA expression of the tumor region. Significant: *P*<0.05. Not Significant: *P*>0.05.

**Supplementary Figure S4.** The correlation between VISTA and various cell subsets and the distribution of VISTA within macrophage subsets.

**A.** Expression correlation between VISTA+ cells and other unique TIICs in tumor region. ns: not significant. **B.** Expression correlation between VISTA+ cells and other unique TIICs in stroma region. ns: not significant. **C.** Density of M1/2-like macrophages within tumor region in GC. **D.** Density of M1/2-like macrophages within stroma region in GC. **E, F.** Comparison of the density of detailed macrophage subtypes among VISTA-positive macrophages in tumor region. ns: not significant. **G, H.** Comparison of the density of detailed macrophage subtypes among VISTA-positive macrophages in stroma region. ns: not significant.

**Supplementary Figure S5.** Comparison of the effective score (A) and effective percent (B) of various tumor-infiltrating immune cells (TIICs) between the VISTA-high group and the VISTA-low group.

**Supplementary Figure S6.** The correlation between the effective score and effective percent of VISTA^+^ macrophages (**A, B**), VISTA^+^ M1-like macrophages (**C, D**), VISTA^+^ M2-like macrophages (**E**) and prognosis.

**Supplementary Figure S7.** Cell annotation and marker expression.

**A.** The distribution of cells from 17 samples. **B.** Cells were classified into 27 clusters. **C.** The ROUGE score of different cell subtypes. **D.** The expression of marker genes in all clusters.

 **Supplementary Figure S8.** The gene expression in different cell subtypes.

**A.** The different expression genes (DEGs) in different cell subtypes. **B.** The marker genes expression in UMAP.

**Supplementary Figure S9.** Secondary clustering of cell subtypes.

**A.** The function subtypes in different cell types. **B.** The marker genes expression of Secondary clustering subtypes.

**Supplementary Figure S10.** The expression atlas of VSIR in different cell subtypes.

**A.** The expression of various immune checkpoints and therapeutic targets across different cell subtypes. **B.** The violin plot showing VSIR expression in different cell subtypes. **C.** The distribution of VSIR expression. Blue dash-line: the cut-off defining VSIR^+^ monocyte-macrophages (MoMacs) and VSIR^-^ monocyte-macrophages (MoMacs).

**Supplementary Figure S11.** The pseudotime trajectory analysis of monocyte-macrophage (MoMac) cells.

**A.** Changes in M1/2-related genes along pseudotime. **B.** Visualization of MoMac cell differentiation trajectories using the Slingshot package based on UMAP. **C.** Changes in M1/2-related genes along pseudotime based on Slingshot package.

**Supplementary Figure S12.** The pseudotime trajectory analysis of CD8^+^ T cells and CD4^+^ T cells.

**A.** Pseudotime trajectory depicting the dynamics of CD8^+^ T cells. **B, C.** Distribution of multiple CD8^+^ T cell subtypes along the pseudotime trajectory. **AD** Pseudotime trajectory depicting the dynamics of CD4^+^ T cells. **E, F.** Distribution of multiple CD4^+^ T cell subtypes along the pseudotime trajectory. **G.** Visualization of CD8^+^ T cell differentiation trajectories using the Slingshot package based on UMAP. **H.** Visualization of CD4^+^ T cell differentiation trajectories using the Slingshot package based on UMAP.

**Supplementary Figure S13.** Comparison of the TME between the VSIR-high group and the VSIR-low group.

**A.** VISR expression levels in monocyte-macrophages (MoMac) across different samples, with the mean value of VSIR in MoMacs annotated above each sample's horizontal line. **B-D.** Comparison of the proportion of all cell subsets, T & NK cell subsets, and myeloid cell subsets between the VISR-high group and the VISR-low group.

**Supplementary Figure S14.** VISR-related spatial transcriptomics analysis.

**A.** Getis-Ord Gi* hotspots of VSIR in ST1, showing that VSIR exhibits a clustered distribution.. **B.** The correlation coefficient between C3^+^ macrophages and T cell distribution. **C.** Enrichment results of galectin-related pathways in ST1. **D.** Differential expression gene (DEGs) in co-localized regions of C3^+^ macrophages and T cells compared to non-co-localized regions. **E-K.** Results of repeated spatial transcriptomics analysis in the ST2 sample. **L-R.** Results of repeated spatial transcriptomics analysis in the ST2 sample.
